# Exercise-augmented THSD7B exhibited a positive prognostic implication and tumor-suppressed functionality in pan-cancer

**DOI:** 10.3389/fimmu.2024.1440226

**Published:** 2024-08-05

**Authors:** Zhiwen Luo, Jinguo Zhu, Zhengyuan Fang, Rui Xu, Renwen Wan, Yanwei He, Yisheng Chen, Shuo Chen, Qing Wang, Qizhi Liu, Shiyi Chen

**Affiliations:** ^1^ Department of Sports Medicine, Huashan Hospital, Fudan University, Shanghai, China; ^2^ Department of Orthopaedics, Nantong Tongzhou Hospital of Traditional Chinese Medicine, Tongzhou, Jiangsu, China; ^3^ The First Affiliated Hospital of Dalian Medical University, Dalian Medical University, Dalian, Liaoning, China; ^4^ The First Clinical Medicine College, Nanjing Medical University, Nanjing, China; ^5^ Internal Medicine of Chinese Medicine, Nanjing Hospital of Chinese Medicine affiliated to Nanjing University of Chinese Medicine, Nanjing, China; ^6^ Department of Orthopaedics, Kunshan Hospital of Chinese Medicine, Kunshan, Jiangsu, China

**Keywords:** physical exercise, breast cancer, THSD7B, pan-cancer, bioinformatics, proliferation, immunotherapy

## Abstract

**Background:**

Breast cancer, one of the most prevalent malignancies among women worldwide, has rising incidence rates. Physical activity, particularly exercise, has emerged as a significant modifier of cancer prognosis, influencing both tumor biology and patient outcomes.

**Methods:**

In this study, we utilized a murine breast cancer model, dividing mice into a control group and an exercise group; the latter underwent 21 days of voluntary running. We conducted RNA sequencing, bioinformatics analysis, pan-cancer analysis, and cellular experiments to investigate the underlying mechanisms influenced by exercise.

**Results:**

Exercise led to a significant reduction in tumor size and weight. Post-exercise mRNA sequencing indicated a notable upregulation of THSD7B in the exercised mice, with significant alterations observed in pathways such as MicroRNAs in cancers and the Calcium signaling pathway. In a broader cancer context, THSD7B showed considerable expression variability, being significantly downregulated in several cancers, correlating with positive prognostic outcomes in PRAD, LAML, KIRC, and GBM and highlighting its potential role as a prognostic marker and therapeutic target. THSD7B expression was also negatively associated with processes of breast cancer cell proliferation, migration, and invasion.

**Conclusion:**

This study underscores the dual role of exercise in modulating gene expression relevant to tumor growth and highlights the potential of THSD7B as a therapeutic target in cancer. Future research should further explore the specific mechanisms by which exercise and THSD7B influence cancer progression and develop immunotherapy-enhanced strategies to change patient outcomes in clinical settings.

## 1 Introduction

Breast cancer, one of the most prevalent malignancies among women, continues to see rising incidence rates globally (1, 2). According to the World Health Organization, it ranks as a leading cause of cancer-related fatalities among women worldwide (3). The disease’s impact is not only profound on health, as it aggressively invades nearby tissues and metastasizes through the lymphatic and circulatory systems to distant vital organs like the bones, liver, lungs, and brain, which significantly complicates treatment strategies and increases their complexity (4–9). Moreover, the socioeconomic effects of breast cancer are substantial, often resulting in significant financial strain during prolonged treatment periods and adversely affecting family and social dynamics due to the psychological burden associated with the illness (10, 11). Consequently, there is an urgent need to enhance our understanding of breast cancer’s underlying biological mechanisms and to develop new, more effective targeted treatments (12, 13).

The positive effects of physical activity on health and cancer prevention are extensive (14, 15). Engaging in regular exercise improves cardiovascular function and muscular strength, enhances bone density, and plays a crucial role in preventing osteoporosis (16, 17). Metabolically, it boosts the body’s energy expenditure, helping to maintain a healthy weight and physique. From an immunological perspective, physical activity elevates lymphocyte counts, fortifying the immune system’s capacity to combat diseases, including cancer (18). Additionally, exercise is effective in reducing psychological and alleviating symptoms of anxiety and depression (19), which contributes to improved mood and overall well-being, indirectly lowering the risk of cancer. Consistent physical activity has been demonstrated to be associated with reduced cancer incidence, possibly due to its role in enhancing antioxidative mechanisms and facilitating the rapid elimination of carcinogens (20–22). Cutting-edge research has further highlighted the therapeutic potential of exercise in the field of oncology. A pivotal study by Luo et al. demonstrated how physical activity could alter the immune landscape of non-small cell lung cancer, shifting it from an immunologically “cold” to a “hot” state (23). This shift suggests that exercise not only boosts the presence of CD8+ T cells and M1 macrophages but also diminishes immunosuppressive cell populations, thus potentiating the efficacy of immunotherapy. The transformative influence of exercise underscores its potential as a valuable complement to traditional cancer therapies, proposing that integrating physical activity into treatment protocols could significantly improve therapeutic outcomes (24–26).

The role of exercise in modulating gene expression has garnered significant attention in recent years, particularly in the context of cancer. Numerous studies have demonstrated that physical activity can influence the expression of various genes involved in cancer progression and response to treatment. For instance, exercise has been shown to modulate the expression of genes related to apoptosis, DNA repair, and immune response, thereby potentially impacting tumor growth and metastasis. For example, exercise such as long-distance running activates oncogenes such as p53 in mouse liver cells. In this paper we used sequencing data focusing on the THSD7B gene that is most significantly altered after exercise.

THSD7B encodes for the thrombospondin type 1 domain-containing protein 7B, a membrane component that plays a critical role in actin cytoskeleton reorganization and is involved in post-translational modifications and glycosylation of proteins (27). Mutations in the THSD7B gene may inhibit signaling pathways related to cell death while enhancing pathways associated with invasion, metastasis, and downregulating immune response pathways, potentially affecting the prognosis of patients with small cell lung cancer (27, 28). THSD7B is associated with cell adhesion, and THSD7B is involved in angiogenesis and oncogenic activities, hence mutations in these genes are frequently observed during cancer progression. Heterozygous variations in THSD7B can lead to reduced cell adhesion, while concurrently increasing the invasiveness and metastatic potential of tumor cells (29, 30).

This research has pinpointed THSD7B as a pivotal target through gene sequencing of mice engaged in voluntary running wheel exercises, positioning it as a strategy against breast cancer. By delving deeper into THSD7B using bioinformatics and cellular biology experiments, this study seeks to elucidate its role in tumor development and progression. Our findings illuminate the mechanisms by which THSD7B influences cancer biology and highlight the potential of exercise-induced molecular responses as a proactive measure in the prevention and treatment of cancer.

## 2 Materials and methods

### 2.1 Cell culture

The 4T1 mouse cancer cell line (catalog KGG2224-1) and MDAMB231 (catalog KGG3220-1) were procured from KeyGEN (Nanjing, China). MDA-MB-468 was procured from FengHui ShengWu, China. 4T1 cells were cultured in RPMI-1640 medium enriched with 10% fetal bovine serum (FBS) and sustained at 37°C in either an ambient atmosphere or one containing 5% CO_2_. MDAMB231 and MDAMB468 cells were cultured in the MEM media with 1% non-essential amino acid and 1 mM sodium pyruvate. All media were added with 10% FBS at 37°C with or without 5% CO_2_.

### 2.2 Animal interventions

Female BALB/c mice, aged 5-6 weeks, were sourced from the Shanghai Laboratory Animal Center (SLAC). To develop a model of triple-negative breast cancer (TNBC), we subcutaneously injected 4T1 cells (5 × 10^6) into the abdomens of the mice. The selection of this particular strain and demographic was due to its suitability for breast cancer research and its reliable response to exercise interventions. Each mouse’s health was confirmed by a veterinarian before the study began. The mice were kept in a controlled setting with a 12-hour light/dark cycle and had unrestricted access to food and water. We stratified the mice into two cohorts: an exercise group (E) and a non-exercise group (NE), with each group containing five mice. Over a 21-day period, the exercise group engaged in voluntary running, while the non-exercise group was kept under standard care conditions without dietary limitations (no speed or distance limitation). Following the 21-day period, all mice were humanely euthanized, and their tumor tissues were harvested for subsequent mRNA sequencing analysis. Animal experiments were granted by Ethics Committees at Nanjing Medical University (IACUC-2312041).

### 2.3 mRNA sequencing and bioinformatics analysis

Twenty-one days after initiating treatment, tumor samples were meticulously collected from mice for mRNA sequencing analysis. Subsequently, cell samples from these treatments were carefully harvested. Total RNA extraction from these samples was conducted using the esteemed RNeasy Mini Kit (Qiagen, Hilden, Germany). Following RNA extraction, the construction of paired-end libraries was meticulously performed using the TruSeq RNA Sample Preparation Kit (Illumina, USA), strictly following the protocol outlined by TruSeq (31). The responsibility for constructing and sequencing these libraries was entrusted to the Shanghai Biotechnology Corporation. We have uploaded the raw sequencing results to a public cloud storage space “NutCloud” as requested. The access link is below: https://www.jianguoyun.com/p/DR7owysQv4LSDBiz5sQFIAA.

For accurate mapping of clean reads to the Rnor 6.0 reference genome, with an allowance for up to two mismatches, the highly acclaimed Hisat2 software (version 2.0) was employed (32). After genome mapping, the revered Stringtie software (version 1.3.0) was used to generate and annotate Fragments per kilobase of exon per million (FPKM) values. Statistical significance was established with a P-value threshold set according to the false discovery rate (FDR). mRNAs demonstrating a fold change of ≥ 2 and an FDR ≤ 0.05 were classified as differentially expressed.

To further elucidate the biological pathways impacted by the treatments, an exhaustive KEGG pathway analysis was conducted using the esteemed KEGG database (http://www.genome.ad.jp/kegg) within the R environment (33). Additionally, Gene Set Enrichment Analysis (GSEA) was carried out using the R BiocManager to provide deeper insights into the molecular mechanisms affected by the treatments.

### 2.4 Pan-cancer analysis

#### 2.4.1 Gene expression and datasets obtained

We employed the Human Protein Atlas (HPA) to aggregate extensive RNA and protein expression profiles of THSD7B from human samples (34). Additional insights into THSD7B expression across various tissues and cell lines were derived from the Harmonizome database. To further enrich our analysis, we integrated mRNA expression data for THSD7B from cancerous, paracancerous, and normal tissues sourced from the TCGA and GTEx databases. Our examination covered a broad spectrum of 33 cancer types, including, Adrenocortical carcinoma (ACC), Bladder Urothelial Carcinoma (BLCA), Breast invasive carcinoma (BRCA), Cervical squamous cell carcinoma and endocervical adenocarcinoma (CESC), Cholangiocarcinoma (CHOL), Colon adenocarcinoma (COAD), Lymphoid Neoplasm Diffuse Large B-cell Lymphoma (DLBC), Esophageal carcinoma (ESCA), Glioblastoma multiforme (GBM), Head and Neck squamous cell carcinoma (HNSC), Kidney Chromophobe (KICH), Kidney renal clear cell carcinoma (KIRC), Kidney renal papillary cell carcinoma (KIRP), Acute Myeloid Leukemia (LAML), Brain Lower Grade Glioma (LGG), Liver hepatocellular carcinoma (LIHC), Lung adenocarcinoma (LUAD), Lung squamous cell carcinoma (LUSC), Mesothelioma (MESO), Ovarian serous cystadenocarcinoma (OV), Pancreatic adenocarcinoma (PAAD), Pheochromocytoma and Paraganglioma (PCPG), Prostate adenocarcinoma (PRAD), Rectum adenocarcinoma (READ), Sarcoma (SARC), Skin Cutaneous Melanoma (SKCM), Stomach adenocarcinoma (STAD), Testicular Germ Cell Tumors (TGCT), Thyroid carcinoma (THCA), Thymoma (THYM), Uterine Corpus Endometrial Carcinoma (UCEC), Uterine Carcinosarcoma (UCS), Uveal Melanoma (UVM).

The analytical phase of our study utilized R software (version 4.2.2) with the ggplot2 package to visualize THSD7B expression across these diverse cancer types. We defined the median expression level as the cutoff for determining differential expression. To analyze differences between expression levels among groups, we employed the Wilcoxon rank-sum test, allowing us to rigorously evaluate the expression patterns of THSD7B and their statistical significance across various oncological contexts.

#### 2.4.2 Survival analysis of THSD7B in the 33 cancers

To assess the prognostic significance of THSD7B expression in a variety of cancers, we performed survival analysis using the “survival” package in R (35). Kaplan-Meier curves were generated and Cox regression models were applied to examine differences in survival rates between patient groups characterized by high versus low THSD7B expression levels. The effects of THSD7B on patient survival were depicted in forest plots, constructed with the help of the “survminer” and “ggplot2” packages.

#### 2.4.3 Genetic alteration analysis of THSD7B

We explored the genetic alterations of THSD7B by leveraging data from cBioPortal. Our study detailed the frequencies of somatic mutations and provided comprehensive genomic information, shedding light on the mutation spectrum of THSD7B across various cancer contexts.

#### 2.4.4 Immunogenomic analyses of THSD7B in the 33 cancers

Our immunogenomic analysis spanned 33 different cancer types, utilizing the “GSVA” package and “ssGSEA” algorithm to evaluate the associations between THSD7B expression and a range of immune components, such as tumor-infiltrating lymphocytes, immunostimulators, immunoinhibitors, MHC molecules, chemokines, and chemokine receptors (36). We determined the correlations using Spearman’s correlation coefficient, considering p-values below 0.05 as statistically significant. The relationships were visually represented in heatmaps created using the “ggplot2” package, providing a clear and comprehensive depiction of the immune landscape influenced by THSD7B expression.

#### 2.4.5 Functional enrichment analysis of THSD7B

We conducted Gene Ontology (GO) and Kyoto Encyclopedia of Genes and Genomes (KEGG) pathway enrichment analyses to explore the biological functions and pathways linked to genes that interact significantly with THSD7B. These interacting genes were pinpointed using the STRING database and assessed via the “clusterProfiler” and “org.Hs.eg.db” packages in R. A rigorous cutoff threshold of p-value < 0.01 was applied to both GO and KEGG enrichment analyses to ensure statistical significance. The results of these analyses were visually depicted using bubble charts, which were crafted using the “ggplot2” package in R.

### 2.5 Cellular experiments

#### 2.5.1 Silencing of THSD7B gene expression

To silence the THSD7B gene in tumor cells, small interfering RNAs (siRNAs) specifically designed to target THSD7B mRNA were synthesized (32). These siRNAs were algorithmically optimized for effective gene targeting. The silencing process involved the following steps: siRNA Transfection: Cells were transfected with siRNA using Lipofectamine 2000 (Invitrogen) according to the manufacturer’s protocol. Briefly, 24 hours post-cell seeding, a mixture of Lipofectamine 2000 and siRNA was prepared to form complexes, which were then introduced to the cells. Evaluation of Knockdown Efficiency: 48 hours following transfection, the reduction in THSD7B mRNA and protein levels was quantified using real-time quantitative PCR (qPCR) to assess the knockdown efficacy.

#### 2.5.2 Overexpression of THSD7B gene

To augment the expression of THSD7B, we engineered a plasmid encoding the full-length coding sequence of THSD7B under the control of the CMV promoter. The overexpression protocol included the following steps: Plasmid Construction: THSD7B cDNA was cloned into the pCMV expression vector. The accuracy of the inserted sequence was confirmed through gene sequencing. Plasmid Transfection: Similar to the siRNA transfection, cells were transfected with the constructed plasmid using Lipofectamine 2000, 24 hours after cell seeding. Verification of Expression: The levels of THSD7B mRNA and protein were measured 48 hours post-transfection by qPCR to validate the effectiveness of the overexpression strategy.

#### 2.5.3 Viability/proliferation/migration/invision

To assess the viability of cancer cells, the cells were cultured in suspension before being plated at a density of 5 × 10^3 cells/mL (100 μL per well) into a 96-well plate, which was then incubated at 37°C. After 24 hours, 10 μL of CCK-8 reagent (catalog KGA9305, KeyGEN, Nanjing, China) was added to each well. The mixture was incubated for an additional two hours, after which the optical density was measured at 450 nm using a microplate reader to determine cell proliferation (37).

For migration and invasion assays, Transwell chambers were used, with migration assays performed without Matrigel coating and invasion assays conducted with a Matrigel coating (38). Cancer cells (5×10^4) in 200 μL of serum-free medium were placed in the upper chamber, while the lower chamber contained 600 μL of medium with 10% FBS to stimulate cell migration and invasion.

To determine levels of cell proliferation, we analyzed the proliferation rate using a 5-Bromo-2′ -deoxyuridine (BrdU) incorporation assay kit (Cell Signaling Technology, MA, USA) following the manufacturer’s instructions (39).

#### 2.5.4 Real-time quantitative polymerase chain reaction

To quantify mRNA levels, total RNA was extracted from cells and tissues using Trizol reagent (Invitrogen) and measured precisely using a Nanodrop instrument (Thermo Scientific, USA) (40). The extracted RNA was then converted into cDNA, which served as the template in qPCR assays conducted with the TB Green™ Premix Ex Taq™ II kit (Takara; RR820A). GAPDH served as the internal reference gene. qPCR primers were synthesized by Bioengineering (Shanghai, China), specifically designed for mRNA amplification. The relative mRNA expression levels were determined by the comparative Ct method (2^-ΔΔCt), ensuring the robustness of our findings through multiple independent experiments. All experimental data were normalized to the control conditions to maintain consistency across measurements. Detailed primer sequences are listed in **Supplementary Table 1**.

### 2.6 Statistical methods

Statistical analysis and figure generation were performed with R language version 4.0.2 and GraphPad Prism 9.0. For the comparison of continuous variables between two groups, the choice between the Student t-test and the Mann-Whitney test depended on specific conditions. When comparing multiple groups, either one-way ANOVA or the Kruskal-Wallis test with subsequent multiple comparisons was used, depending on the circumstances. The prognostic significance of categorical variables was determined using the log-rank test. Statistical significance was set at a P value <0.05 across all analyses.

## 3 Results

### 3.1 Impact of voluntary running on tumor growth and gene expression

**Figure 1:**
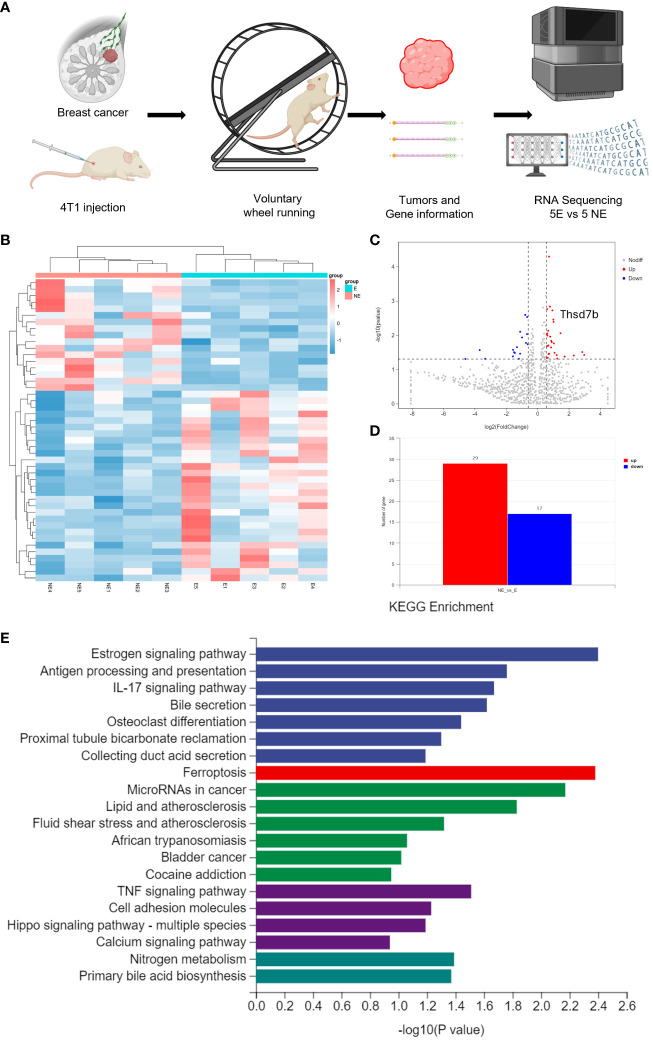
Voluntary wheel running exercise inhibits breast cancer growth. **(A)** Schematic diagram of the experiment. **(B)** Heatmap of Hierarchical clustering analysis of changed mRNAs. **(C, D)** Volcano plot and column of mRNAs differentially expressed between NE and E group. **(E)** Histogram plot showing KEGG enrichment by all the differentially expressed mRNAs expressed in tumors, including biological process, cellular component, and molecular function.

Following the intervention of exercise, a significant reduction in tumor size and weight was observed at day 21, with minimal changes in the body weight of the mice (**Supplementary Figures 1A, B**). We then conducted mRNA sequencing analysis on five matched pairs (**Figure 1A**). The quality control results confirmed normal parameters, with high intra-group consistency and notable expression differences between groups (**Supplementary Figure 2B**). Volcano plots and heatmaps revealed differential expression of 46 genes, among which THSD7B expression was significantly increased in the exercise group (E), representing more than 2 times that in the non-exercise group (NE), with a p-value of 0.009 (**Figures 1B–D**). Gene enrichment analysis highlighted significant alterations in extracellular components, with the most pronounced changes observed in the Ferroptosis, Estrogen signaling pathway, and TNF-α signaling pathway. In details, it is shown that the estrogen signaling pathway, antigen processing and presentation, and IL-17 signaling pathway exhibit the highest significance in the samples studied (-log10(p-value) close to 2.6), while the ferroptosis pathway (-log10(p-value) around 2.1) also exhibits a particularly significant variation, marked in red. Other notable pathways include MicroRNAs in cancer and lipid- and atherosclerosis-related pathways, reflecting the important biological roles and potential research value of these pathways under this experimental condition (**Figure 1E**, **Supplementary Figure 2A**).

### 3.2 Pan-cancer analysis

#### 3.2.1 Expression variability of THSD7B in pan-cancer

**Figure 2:**
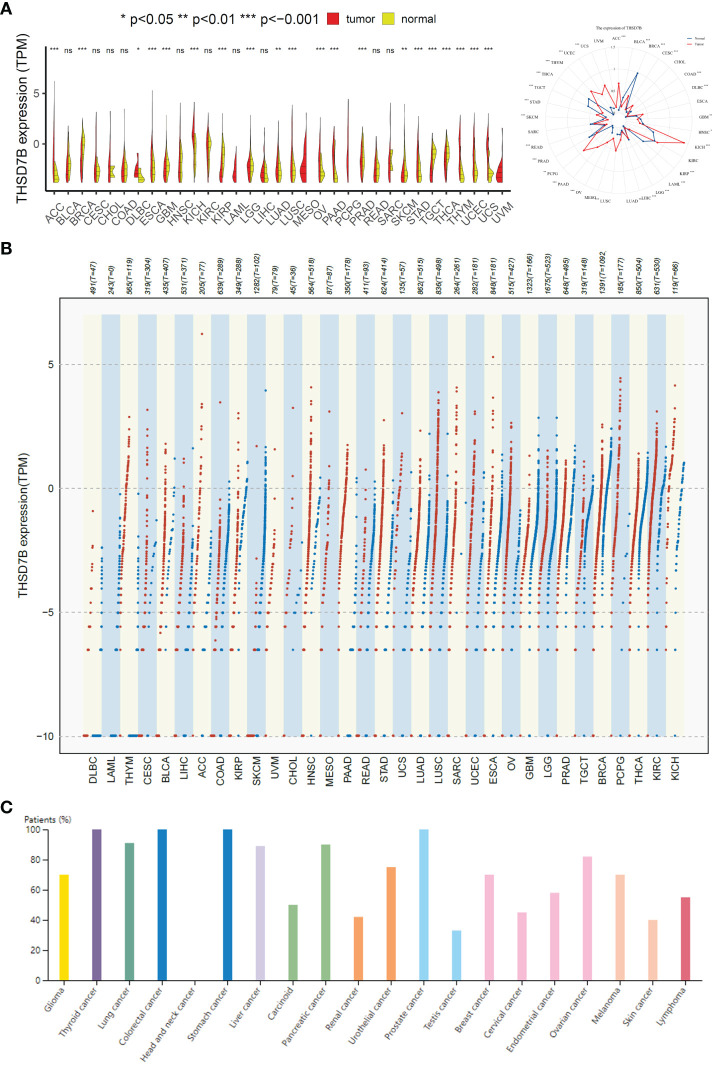
Differential expression pattern of THSD7B. **(A)** Differential THSD7B mRNA expression between unpaired samples in TCGA cancers. The red dot represents cancer samples, and the yellow one represents paired normal samples. Radargrams visualize and compare THSD7B expression in different tumors. *p < 0.05, **p < 0.01, and ***p < 0.001, ns: no significance. **(B)** Differential THSD7B mRNA expression paired samples in TCGA cancers. The red column represents cancer samples, and the blue column represents normal samples. The normal group was normal tissue in TCGA and GTEX databases. **(C)** THSD7B protein expression in different cancer types in Human Atlas.

To evaluate the expression of THSD7B mRNA in normal human tissues, we analyzed data from the GTEx, HAP, and Consensus datasets. In-depth evaluation using RNA-seq data from TCGA and GTEx databases revealed significant expression differences in THSD7B across 33 types of cancer. In unmatched samples (**Figure 2A**, **Supplementary Figure 3A**), THSD7B was notably upregulated in cancers like ACC, KICH, LUSC, OV, PPAD, PCPG, STAD, THYM, UCEC and UCS, and downregulated in BLCA, BRCA, KIRP, PRAD, and THCA. In matched samples (**Figure 2B**, **Supplementary Figure 3B**), upregulation was not significant in all the cancer types, while downregulation was noted in BLCA, BRCA, COAD, KIRC, KIRP, and PRAD. The Human Atlas database further assessed the protein expression of THSD7B across various cancers, showing upregulation in Thyroid cancer, Stomach cancer, Prostate cancer, Liver cancer, Pancreatic cancer, Lung cancer, and Colorectal cancer without significant downregulation in any cancer type (**Figure 2C**).

#### 3.2.2 Prognostic impact of THSD7B in pan-cancer

**Figure 3:**
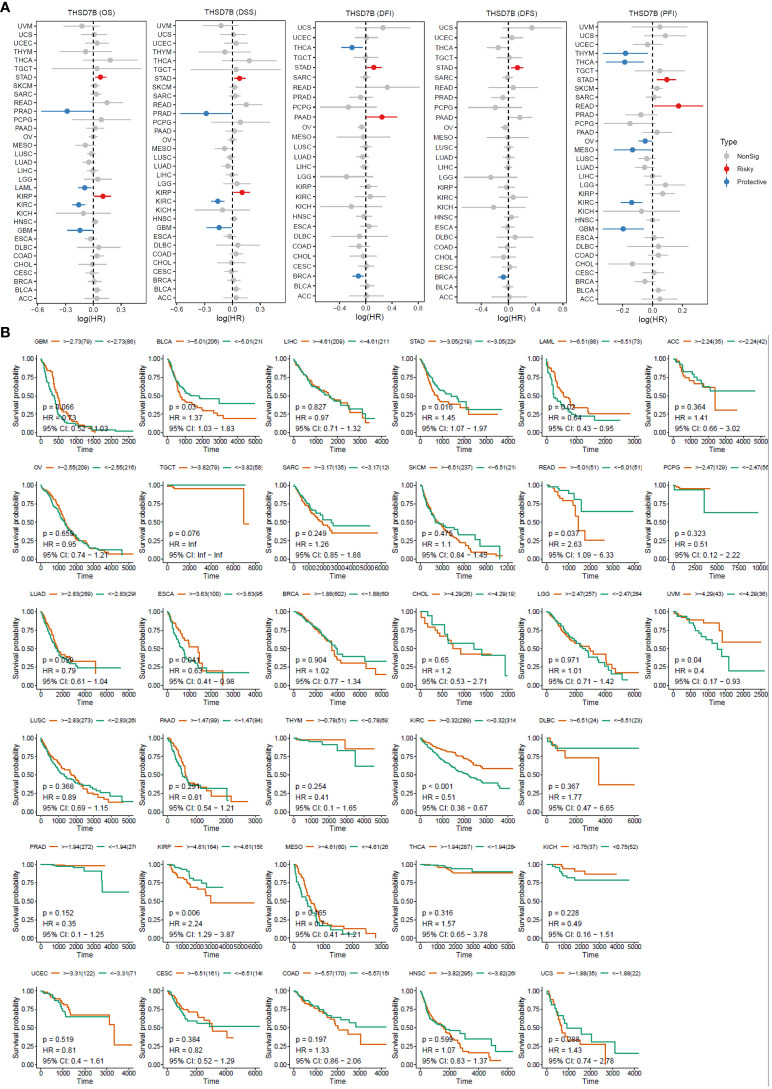
High expression of THSD7B increased patient survival period. **(A)** Forest plot of hazard ratios (HR) for overall survival (OS), PFI, DSS, DFS, and DFI for different cancer types associated with THSD7B expression. Dots indicate log-transformed hazard ratios, red indicates significant risk, blue indicates protective associations, and gray indicates non-significant associations. **(B)** Individual OS figures for each cancer type.

For overall survival (OS) and disease-specific survival (DSS), THSD7B posed a risk factor in STAD and KIRC, while it acted as a protective factor in PRAD, LAML, KIRC, and GBM (**Figures 3A, B**). For disease-free interval (DFI) and disease-free survival (DFS), THSD7B was a risk factor in STAD and a protective factor in THCA and BRCA (**Figure 3A**). For progression-free interval (PFI), THSD7B was a risk factor in STAD and READ, however, it acted as a protective factor in THYM, THCA, OV, MESO, KIRC, and GBM.

#### 3.2.3 Correlation analysis of THSD7B in pan-cancer

**Figure 4:**
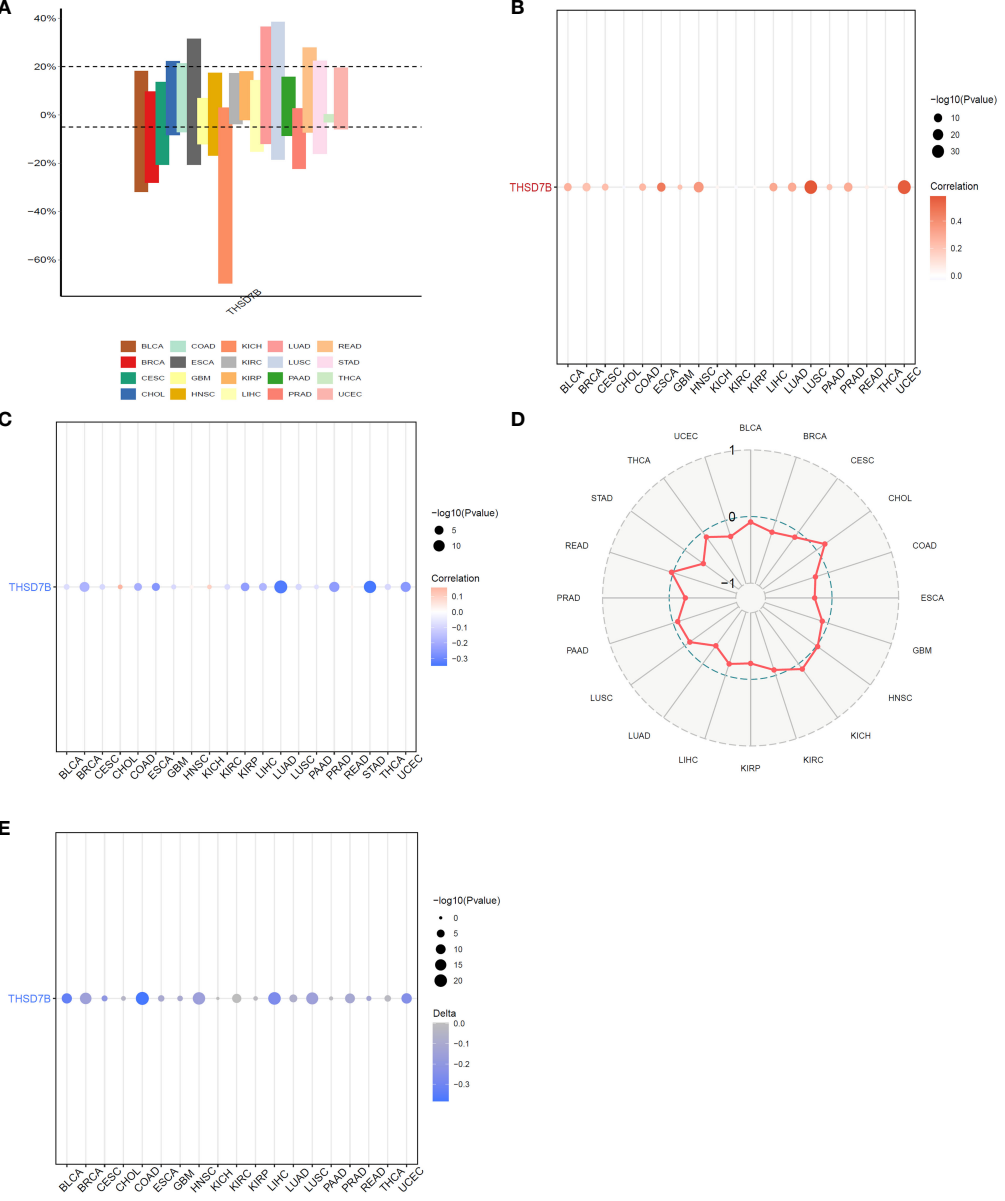
Correlation analysis of THSD7B in pan-cancer. **(A)** Bar graphs illustrate THSD7B copy number variation in different cancers. **(B)** THSD7B copy number and pan-cancer direct correlation analysis. **(C, D)**. The correlation between tumor mutational burden (TMB) and THSD7B expression. **(E)** The correlation between the methylation status of gene promoter regions and THSD7B in multiple cancer types.

Copy number variations (CNVs), a common form of genomic instability in cancer, can lead to altered gene expression affecting cell proliferation, differentiation, and death. Bar graphs (**Figure 4A**) showed changes in THSD7B copy numbers across various cancers, with significant variations in KICH. Further correlation analysis indicated a positive correlation between UCEC and LUSC (**Figure 4B**). Additionally, the relationship between tumor mutational burden (TMB) and THSD7B expression was investigated, revealing a positive correlation in CHOL and KICH, and a negative correlation in BRCA, LUAD, STAD, UCEC, and PRAD (**Figures 4C, D**). Promoter methylation, a critical epigenetic regulatory mechanism affecting gene expression without altering the DNA sequence, was analyzed to explore its relationship with THSD7B expression across multiple cancer types. Both unmatched and matched tumor samples showed a negative correlation between THSD7B expression and methylation, particularly in COAD and LIHC (**Figure 4E**).

#### 3.2.4 Analysis of THSD7B on the immune microenvironment across cancers

**Figure 5:**
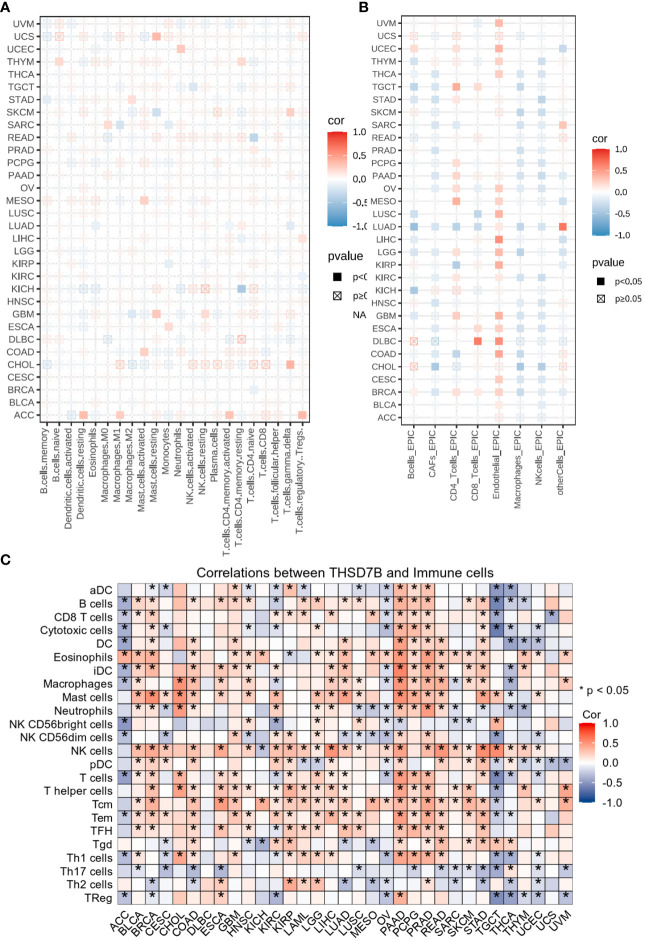
Analysis of immune microenvironmental cellular regulation of pan-cancer by THSD7B. **(A)** Heatmap of immune cell infiltration in pan-cancer analyzed using the Cibersort method. Each cell represents the correlation between THSD7B expression and the level of a specific immune cell type, and the intensity and sign of the color correspond to the strength and direction of the correlation, respectively. Statistical significance is indicated by the box around the cell. **(B)** THSD7B pan-cancer immuno-infiltration analysis using Cibersort. **(C)** Gene Commons data analysis of correlations between single genes and immune infiltration results, using heatmap format to present results. Significance was calculated with Student’s t test. *P < 0.05.

Heatmap analysis from **Figure 5A** intricately details the correlations between THSD7B expression and various immune cell subtypes across different types of cancers. Notably, in cancers such as UCS (Breast Cancer) and GBM (Colorectal Adenocarcinoma), a significant positive correlation exists between THSD7B expression and the rest of mast cells, suggesting that elevated expression of THSD7B may promote mast cell release of immune factors, antigen presentation, and other effects, thereby allowing the tumor to present under immune surveillance, which is conducive to tumor growth and metastasis. Other cells such as, B cells, CD8+ T cells or NK cells are positively correlated with ThSD7B and this correlation is statistically significant (as shown by p<0.05), then this could mean that ThSD7B can promote the function of these immune cells, which may suggest that ThSD7B can improve the tumor microenvironment and promote anti-tumor immunity. Additionally, in most certain cancer types, THSD7B shows a negative correlation with regulatory T cells (Tregs), which play a critical role in modulating the immune system, particularly in maintaining immune tolerance and suppressing excessive immune responses. Increased THSD7B expression might inhibit the functionality of Tregs, thereby fostering an immune-enhanced tumor microenvironment favorable for tumor survival and progression. Conversely, in Lung Adenocarcinoma (GBM), THSD7B exhibits a negative correlation with natural killer (NK) cells, although this association generally lacks statistical significance (**Figure 5B**). This trend implies that in certain cancer contexts, THSD7B expression may inversely affect the immunosurveillance capabilities of NK cells, potentially contributing to mechanisms of immune escape.

EPIC analysis, a vital tool in studying the tumor microenvironment, enables researchers to understand the dynamic variations of different cell types within tumors, which is crucial for advancing tumor immunology and developing new therapeutic strategies. From the heatmap, it is evident that THSD7B’s correlations with various immune cells vary, illustrating the heterogeneity of tumor microenvironments. For instance, in DLBC and LIHC, endothelial cells show a strong positive correlation with THSD7B expression, suggesting their significant role in supporting or enhancing tumor growth and invasion, closely linked with the expression of this gene. Moreover, in cancers like DLBC, the activity of CD8+ T cells significantly correlate with THSD7B expression, reflecting their importance in the tumor immune response and the potential regulatory role of this gene. Further analysis using the TCGA database’s pan-cancer dataset revealed a broadly positive correlation between THSD7B and various immune cells across different cancer types, which suggested THSD7B can be a positive factor for immune microenvironment and immunotherapy (**Figure 5C**).

#### 3.2.5 Pathway enrichment and key gene mutation analysis of THSD7B across cancers

**Figure 6:**
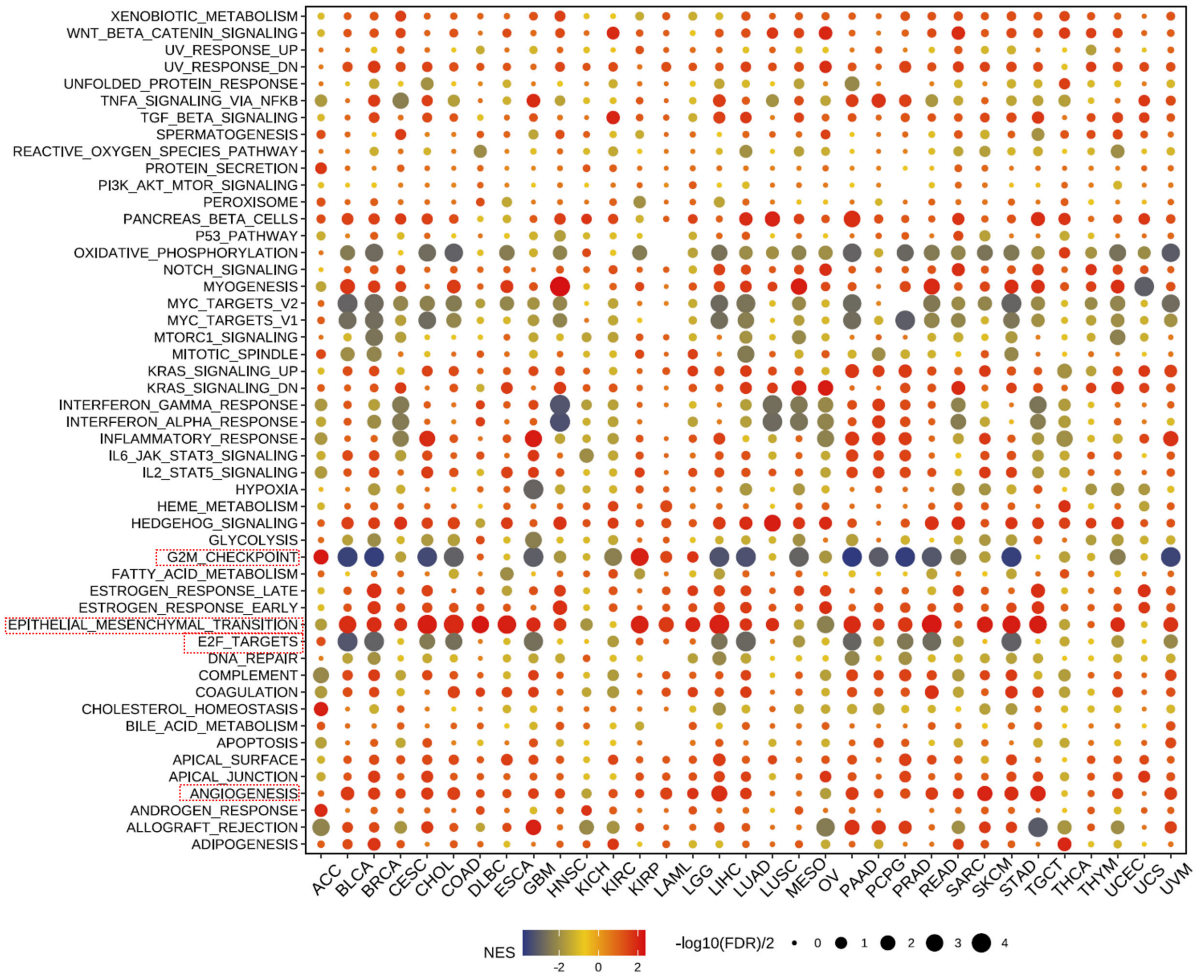
Pathway enrichment of THSD7B and mutational analysis in pan-cancer. Dot plots represent pan-cancer GSEA results using the official immunization gene set (GMT file) as a reference. Functional pathways are from GM7 files and are shown on the y-axis, with different cancer types shown on the x-axis. Dot color indicates correlation with THSD7B expression; red indicates positive correlation and blue negative correlation. The size of the dots represents the -log10(FDR) value, indicating the significance of the enrichment. Analysis of mutation frequency and CNV in TCGA-COAD/READ. The mutation frequency of RNA modification “writers” among 20 cancer types in the TCGA cohort. The horizontal axis represents cancer types, and the number of samples is given in the parentheses. The vertical axis lists the names of the genes.

Our further evaluation of THSD7B’s function in pan-cancer contexts revealed significant findings via the GSEA methodology. THSD7B notably suppresses G2M checkpoint and E2F target pathways, potentially hindering conditions favorable for tumor cell proliferation. Additionally, THSD7B significantly enhances pathways such as angiogenesis and epithelial-mesenchymal transition (EMT), all of which are documented to potentially regulate tumor growth and metastasis (**Figure 6**).

A heatmap depicting the frequency of key gene mutations across various cancers highlights the high mutation rates of genes such as TP53 in LUSC, APC in READ, and PTEN in UCEC, indicating their common involvement in these cancers. Specific cancer types like UCEC, LUAD, and LUSC show frequent mutations in genes like TP53 and CDH10, providing insights that may guide therapeutic strategies (**Supplementary Figure 5A**).

### 3.3 Impact of THSD7B on breast cancer cells

**Figure 7:**
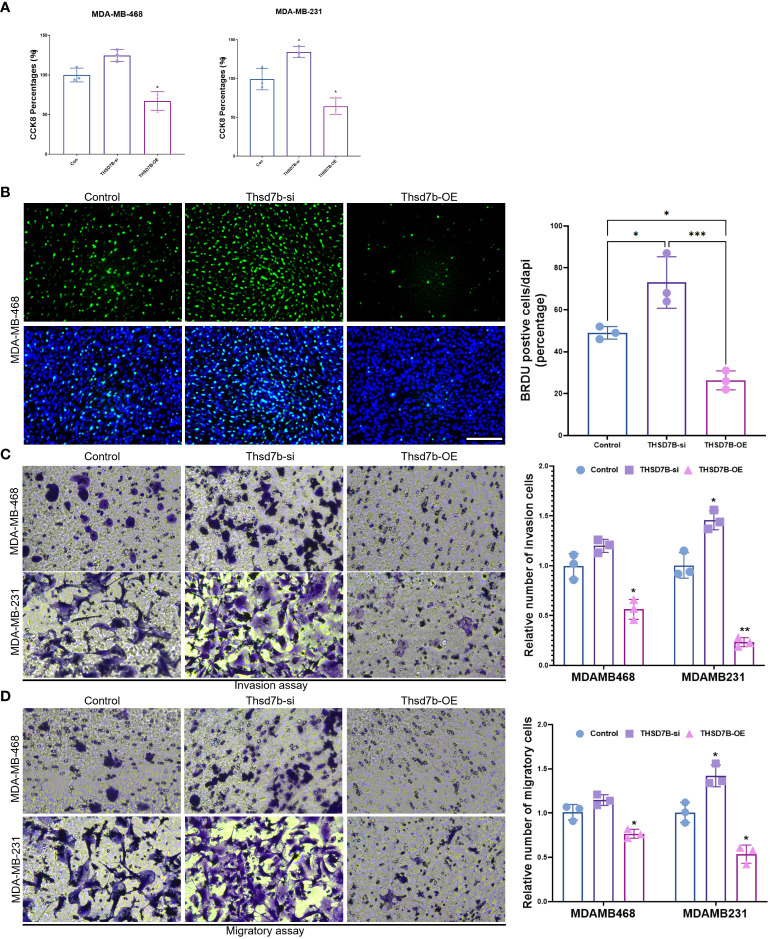
THSD7B promotes breast cancer tumor cell growth. **(A)** The proliferative capacity of control, THSD7B-inhibited, and THSD7B-overexpressed tumor cells was examined at 24h, 48h, and 72h after transfection by CCK-8 assay. Data are presented as mean ± SD. Significance was calculated with student t test. *P < 0.05, **P < 0.01, ***P < 0.001. **(B)** The apoptotic level of control, THSD7B-inhibited, and THSD7B-overexpressed tumor cells was examined at 24h after transfection by flow cytometry. Significance was calculated with student t test. *P < 0.05, **P<0.01. **(C, D)**. The migratory and invasive capacity of control, THSD7B-inhibited, and THSD7B-overexpressed tumor cells were examined at 24h after transfection by Boyden chamber assay. Total original magnification, 200×. Significance was calculated with student t test. **P<0.01, ***P<0.001.

**Figure 8:**
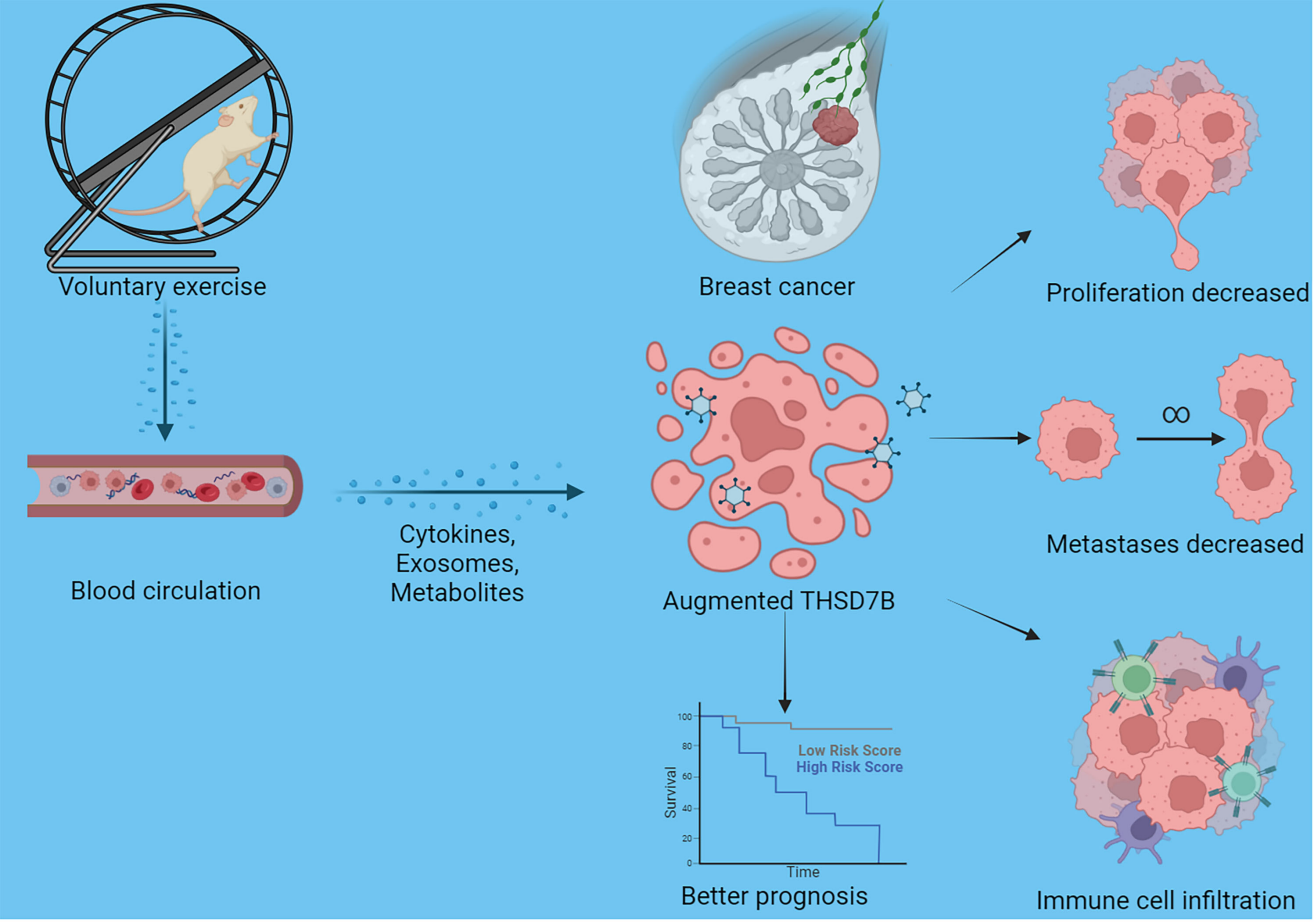
Schematic graph of this study. Exercise increased THSD7B expression of cells in breast cancer through a circulatory effect, which promotes immune cell infiltration, decreased tumor cell metastases/proliferation, and improves the prognosis of tumor patients.

Finally, our study delves into the cellular functions of THDSD7B. We validated the expression of the THDSD7B gene after siRNA or plasmids intervention (**Supplementary Figure 6**). Compared to control cells, overexpression of THDSD7B in MDAMB468 and MDAMB231 breast cancer cells leads to suppressed proliferation and cell viability, while suppression of THDSD7B expression promotes proliferation and cell viability (**Figures 7A, B**). Furthermore, overexpression of THDSD7B significantly inhibits the migratory and invasive capabilities of these tumor cells, whereas its inhibition increases these properties (**Figures 7C, D**). Overall, upregulated targeting THDSD7B could directly inhibit tumor cells, significantly impeding cancer progression and presenting a novel therapeutic target (**Figure 8**).

## 4 Discussion

Our study underscores the formidable challenge posed by breast cancer, a leading cause of cancer-related deaths among women globally (41). Given the disease’s capacity to metastasize and its substantial socioeconomic impact, innovative treatment strategies that extend beyond conventional therapies are urgently needed. In this context, our research provides compelling evidence for the potential of physical activity as an adjunct therapy in breast cancer management, an approach that could revolutionize current treatment paradigms. Our findings indicate that THSD7B may positively affect prognosis and inhibit tumor progression across a range of cancers. Additionally, exercise appears to inhibit breast cancer progression potentially by up-regulating THSD7B.

The results presented in our study illustrate the profound effects of voluntary running on tumor growth and gene expression, particularly highlighting the modulation of the THSD7B gene. This protein, involved in crucial cellular processes such as actin cytoskeleton reorganization, has shown significant overexpression in response to exercise, with the expression levels in the exercise group being more than double those in the non-exercise group. Such findings are critical as they suggest that physical activity can influence cellular functions that are directly linked to cancer progression, such as cell adhesion and metastatic potential (42). Gene enrichment analysis further demonstrated significant alterations in pathways associated with ferroptosis and estrogen signaling—both of which are pivotal in the context of breast cancer. These changes not only provide insights into the molecular mechanisms by which exercise could retard tumor growth but also highlight potential therapeutic targets for future interventions.

The relationship between genes and pan-cancer prognosis in cancer patients could potentially reflect its influence on tumor progression and also facilitate the development of tumor markers (43). Our pan-cancer analysis revealed variable expression of THSD7B across different cancer types, suggesting a complex role in oncogenesis (44). The differential expression patterns observed across various cancers underscore the gene’s potential as a biomarker for cancer progression and prognosis. Notably, upregulation of THSD7B’s in most tumors seems to imply a better prognosis, suggesting that there may be a potential regulatory role of THSD7B on tumor progression and microenvironment. However, THSD7B’s upregulation in aggressive cancers such as ovarian and pancreatic suggests a possible role in promoting malignancy, which could be mitigated by targeted therapies. The prognostic implications of THSD7B expression were evident, with its expression correlating with both risk and protective factors across different cancer types. This duality emphasizes the need for a nuanced understanding of THSD7B’s function in the tumor microenvironment, where it may play different roles depending on the specific cellular context and cancer type. The ability of exercise to inhibit cancer as well has been widely reported, our initial dataset was analyzed from a breast cancer model in mice voluntarily exercising and therefore the significantly high expression of THSD7B was selected for pan-cancer analysis. Thus, the use of THSD7B as a mediator molecule of exercise against cancer can be explained in most models, but in some cancers THSD7B plays a poor role, so the expression pattern of THSD7B cannot be generalized across all cancers, but rather its association with cancer needs to be viewed dialectically. Specifically, in certain cancers, high THSD7B expression may enhance cell proliferation and metastasis, possibly through the activation of specific MicroRNAs. Conversely, in other cancers, THSD7B can inhibit tumor growth by inducing apoptosis and reducing angiogenesis.

Our study also delved into the interactions between THSD7B expression and the immune microenvironment, revealing significant correlations with various immune cell types. In a detailed examination of THSD7B’s interaction with the immune microenvironment, our analysis reveals a nuanced and primarily beneficial role for THSD7B in modulating immune responses across various cancers. THSD7B’s positive correlation with key immune effector cells such as mast cells, B cells, CD8+ T cells, and NK cells supports its function in promoting immune activation and enhancing antitumor activity. This contrasts with the typically suppressive behavior of Tregs, where THSD7B exhibits a negative correlation, potentially reducing their immunosuppressive effects in the tumor milieu. Comparatively, literature on other immune-modulating genes suggests a variable influence on the immune landscape depending on the cancer type and genetic context. For instance, studies like those by Guo et al. on PD-L1 have shown that while some genes suppress immune activity (45), others like THSD7B can facilitate a more robust immune response by altering cell populations and their functional state. Our findings suggest that increasing THSD7B expression could disrupt the usual immune evasion tactics by tumors, thereby making the tumor environment more hostile to cancer progression and more amenable to immunotherapeutic interventions. Moreover, our EPIC analysis indicates that THSD7B’s influence extends beyond just lymphoid cells, impacting the endothelial components within tumors, which could affect tumor angiogenesis and metastasis, aligning with findings from Folkman which highlighted similar impacts by other angiogenic modulators (46). This broad impact of THSD7B underscores its potential as a therapeutic target, with implications for both direct cancer therapy and adjunctive immunotherapy.

Our comprehensive analysis of THSD7B’s function in cancer through GSEA has elucidated its complex role in modulating key cellular pathways that influence tumor behavior. Notably, THSD7B appears to actively suppress the G2M checkpoint and E2F target pathways (38, 47), which are crucial for cell cycle progression and DNA replication. This suppression could impede the conditions that typically favor rapid tumor cell proliferation, suggesting a potential tumor suppressor role for THSD7B in these regulatory pathways. Conversely, THSD7B significantly enhances the angiogenesis and epithelial-mesenchymal transition (EMT) pathways. These pathways are essential for tumor growth and metastasis, as angiogenesis facilitates the tumor’s blood supply, and EMT contributes to tumor cell dissemination (48–50). The dual role of THSD7B, both inhibiting and promoting cancer progression through different pathways, underscores the gene’s potential as a multifaceted target for therapeutic intervention. Additionally, our mutation analysis across various cancers has revealed a pattern of frequent mutations in genes such as TP53, APC, and PTEN, particularly in cancers like LUSC, READ, and UCEC. These genes are known for their pivotal roles in regulating cell growth, apoptosis, and genomic stability (51, 52). The prevalence of mutations in these genes, alongside alterations in THSD7B, provides valuable insights into the molecular landscape of these cancers and could inform the development of targeted treatment strategies that address these specific genetic alterations.

From a mechanistic point of view, the upstream signaling molecules that exercise regulates THSD7B and how THSD7B regulates pathways such as estrogen signaling pathway, antigen processing and presentation, IL-17 signaling pathway, ferroptosis pathway, MicroRNAs in cancer, and lipid- and atherosclerosis-associated pathways have not been explored in this paper. Interestingly we searched a range of literature and found that no direct link has been reported for the THSD7B pathway, which needs to be further explored in future studies. In addition, a large body of literature reports that exercise brings about a series of physiological changes, including changes in related molecules in the metabolome, proteins, and genome (53–55). Specifically, we hypothesized that exercise-induced changes in systemic factors (e.g., serum circulating exosomes, muscle-derived cytokines, and hormones) may affect transcription factors, such as the known gene transcription regulators NF-κB and STAT3 (56–59). In addition, the roles of epigenetic modifications (including DNA methylation and histone acetylation) in the regulation of gene expression in physical activity could also affect THSD7B expression (57, 60–62). Furthermore, the direct upstream transcription factors by which exercise regulates THSD7B expression in tumor cells remain unknown. We propose that exercise activates AMP-activated protein kinase (AMPK) and peroxisome proliferator-activated receptor gamma coactivator 1-α (PGC-1α), which are central to tumor cell expression (63, 64). These molecules may affect transcription factors and co-regulators that control THSD7B expression. In addition, exercise regulates the expression of cellular miRNAs, which may post-transcriptionally regulate THSD7B (65, 66).

In addition, reports on THSD7B in the field of oncology are limited, with few explorations of its specific anti-cancer mechanisms. Only one article reported that mutation of THSD7B in patients inhibits cell death-related pathways, up-regulates cell invasion and metastasis pathways, and down-regulates immune response pathways, which ultimately leads to a poor prognosis in lung cancer (27). And in our study stem also found that THSD7B overexpression can inhibit tumor value-added invasion, and vice versa inhibition of its expression leads to stronger tumor cell value-added invasion. In addition, THSD7B also significantly inhibited the expression of immunosuppressive cells, suggesting its potential tumor immune microenvironment regulation function. In this process of THSD7B up-regulation, many downstream pathways will have a series of effects, and the specific mechanism needs to be confirmed by further studies.

In general, the use of specific therapeutic modalities to enhance THSD7B expression (e.g., material carrier-targeted delivery of THSD7B agonists, etc.) holds great promise for tumor-targeted therapy and has clinical translational value (67, 68). Finally, we can also consider THSD7B as a genetic marker to cluster tumor patients and predict their prognosis. In addition, we can use the intervention of exercise prescription for patients with low expression of THSD7B to improve their response to immunotherapy. Implementation of exercise interventions as cancer therapy involves addressing individual differences, designing effective exercise programs, and ensuring patient compliance. We recommend that multicenter randomized controlled trials be conducted to assess the actual effects of exercise interventions in cancer patients. To explore the therapeutic potential of exercise-induced THSD7B expression, we propose the following research directions: Mechanistic studies: To further elucidate the molecular mechanisms by which THSD7B interacts with other pathways and genes in different cancer types (69–71). Clinical trials: Design and implement large-scale, long-term clinical trials to evaluate the effects of exercise interventions on cancer progression and patient prognosis (72, 73). Personalized medicine: Develop personalized exercise programs based on individual cancer types and THSD7B expression levels to maximize efficacy (74, 75).

Our study on the impact of exercise on breast cancer and the role of THSD7B gene expression, while insightful, has limitations. It primarily relies on animal models and *in vitro* experiments, which may not fully replicate human cancer biology (64). The focus on a single gene, THSD7B, might not capture the complexity of cancer’s multifactorial nature. While murine models are invaluable in cancer research, they have inherent differences in immune response and metabolism compared to humans. Therefore, our results need validation in larger-scale human studies. Additionally, the exercise protocol used does not reflect the diversity of human exercise habits, and the findings’ clinical applicability requires validation in diverse human populations through prospective studies to confirm THSD7B’s prognostic and therapeutic potential (16).

## 5 Conclusions

In conclusion, our findings advocate for the integration of physical activity into breast cancer treatment regimes, emphasizing its potential to modulate key genes and pathways involved in tumor progression. The insights gained into the role of THSD7B across various cancers further enhance our understanding of its potential as a therapeutic target, paving the way for more effective and personalized cancer treatments.

## Data availability statement

TThe original contributions presented in the study are publicly available. This data can be found here: GEO, GSE272213 (https://www.ncbi.nlm.nih.gov/geo/query/acc.cgi?acc=GSE272213).

## Ethics statement

The animal study was approved by Ethics Committees at Nanjing Medical University (IACUC-2312041). The study was conducted in accordance with the local legislation and institutional requirements.

## Author contributions

ZL: Conceptualization, Data curation, Formal Analysis, Funding acquisition, Investigation, Methodology, Project administration, Resources, Software, Supervision, Validation, Visualization, Writing – original draft, Writing – review & editing. JZ: Investigation, Methodology, Project administration, Resources, Writing – original draft. ZF: Investigation, Writing – original draft, Writing – review & editing. RX: Data curation, Formal Analysis, Investigation, Writing – original draft. RW: Data curation, Formal Analysis, Investigation, Writing – original draft, Writing – review & editing. YH: Investigation, Writing – original draft, Writing – review & editing. YC: Investigation, Writing – original draft, Writing – review & editing. SC: Investigation, Writing – original draft, Writing – review & editing. QW: Funding acquisition, Resources, Software, Supervision, Validation, Writing – original draft, Writing – review & editing. QL: Funding acquisition, Project administration, Resources, Supervision, Validation, Writing – original draft, Writing – review & editing. SYC: Funding acquisition, Project administration, Resources, Supervision, Validation, Writing – original draft, Writing – review & editing.
