# Supplementary material for: Exercise-augmented THSD7B exhibited a positive prognostic implication and tumor-suppressed functionality in pan-cancer
Source: Front Immunol. 2024 Aug 5;15:1440226. doi: 10.3389/fimmu.2024.1440226 (PMC11330788; doi:10.3389/fimmu.2024.1440226)
Supplement: Supplementary file 1 [file DataSheet_1.docx]

Supplementary Material

# Supplementary Figures and Tables

## Supplementary Figures


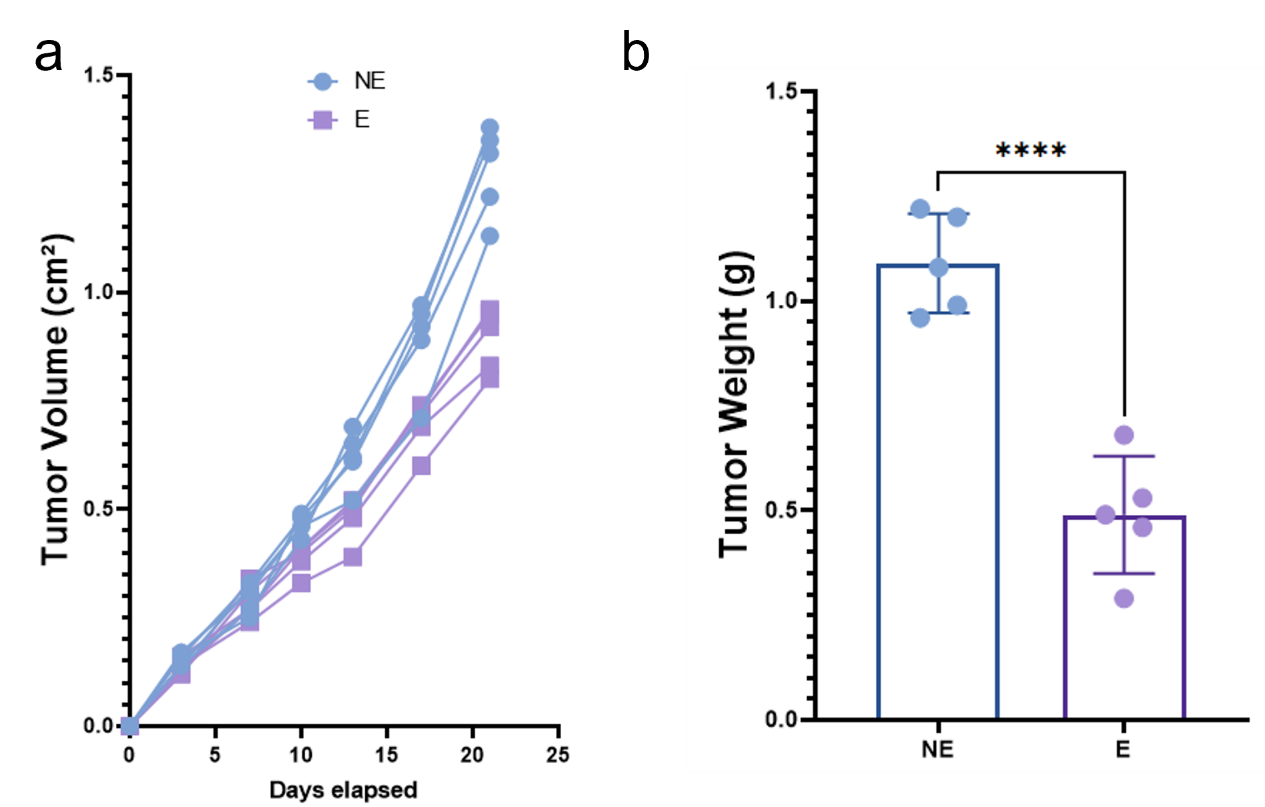


**Supplementary Figure 1.** a. Effect of THSD7B on tumor volume in balb/c mice bearing 4T1 cells. b. Effect of THSD7B on tumor weight in balb/c mice bearing 4T1 cells and quantitative analysis. Data presented as mean ± SD. Significance was calculated with Student’s t test. ***P < 0.001.


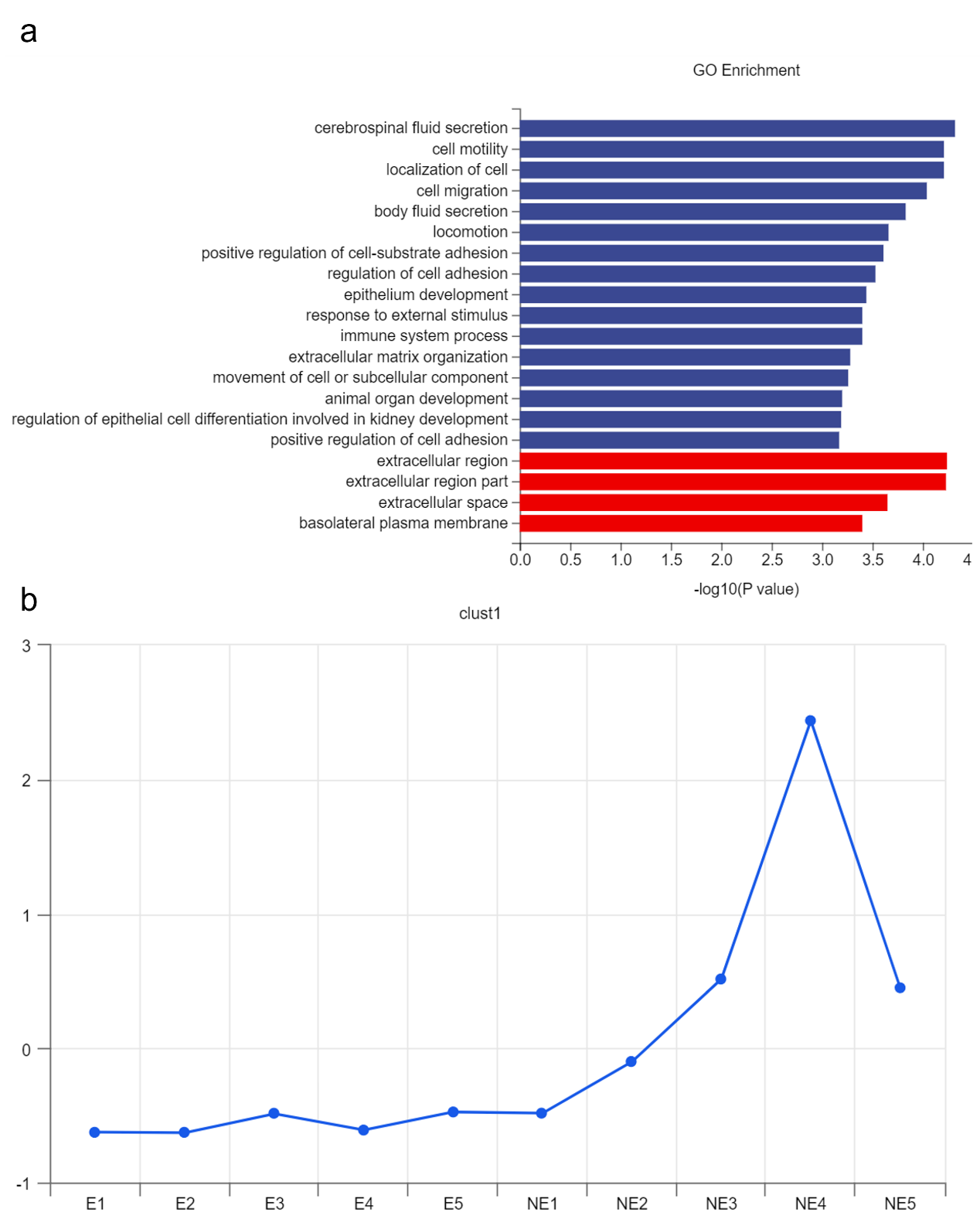


**Supplementary Figure 2**. a. Histogram plot showing GO enrichment by all the differentially expressed mRNAs expressed in tumors, including biological process, cellular component, and molecular function. b. Comparison of gene expression levels between different groups.


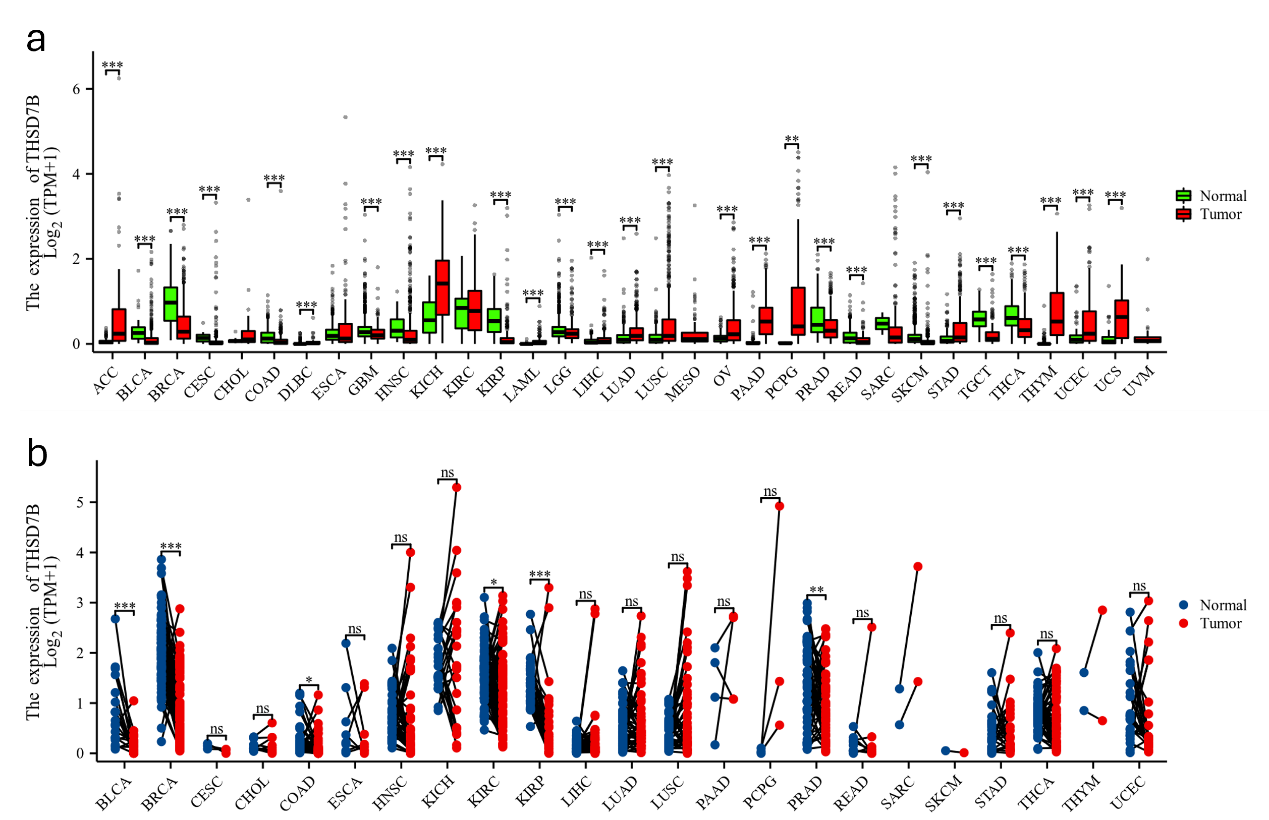


**Supplementary Figure 3.** a-b. THSD7B mRNA expression in normal or tumor tissues from different datasets.


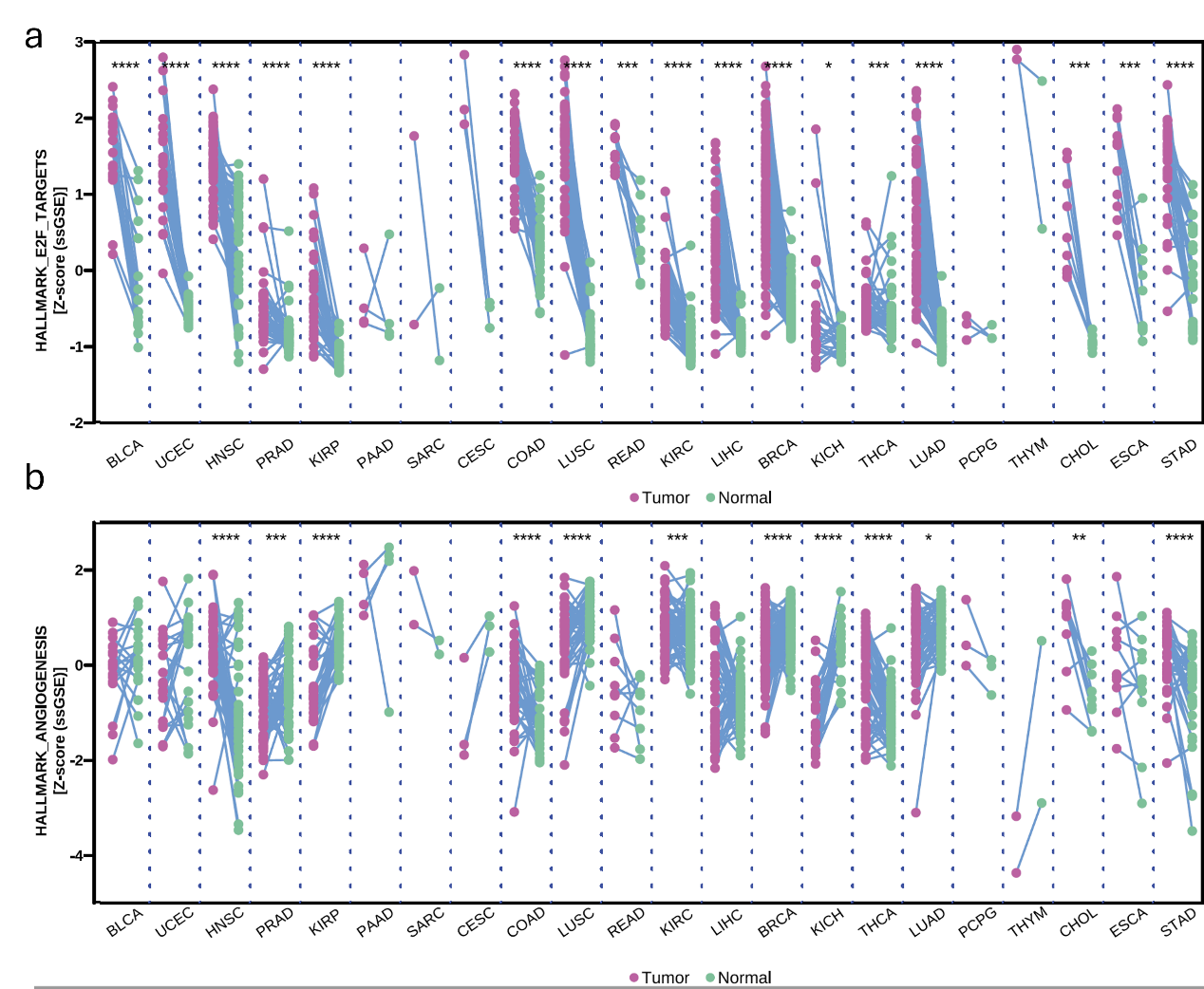


**Supplementary Figure 4**. (a-b) Pairwise comparison plots demonstrate GSEA results for specific pathways, comparing gene set enrichment in tumor versus normal tissue samples. y-axis indicates the normalized enrichment score (NES) and x-axis categorizes cancer type. Color fills indicate the density of data points, with pink indicating tumors and cyan indicating normal tissue distribution.


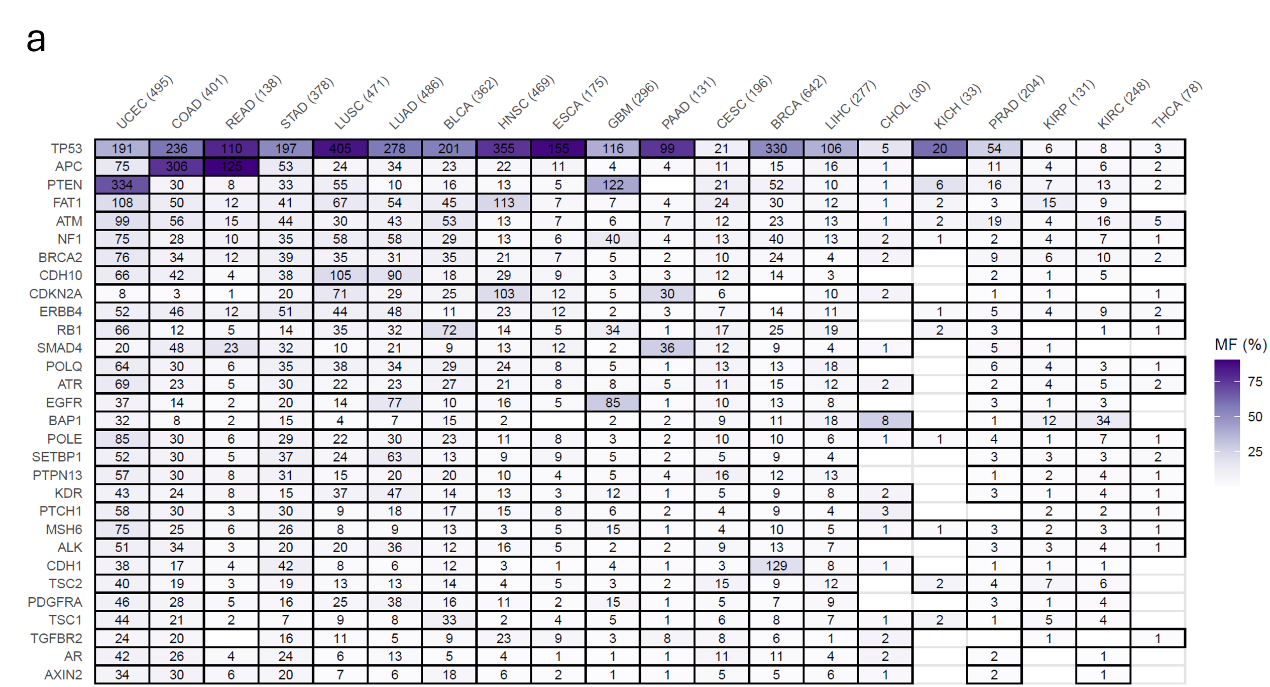


**Supplementary Figure 5**. **Mutational analysis in pan-cancer** (a). Analysis of mutation frequency and CNV in TCGA-COAD/READ. The mutation frequency of RNA modification “writers” among 20 cancer types in the TCGA cohort. The horizontal axis represents cancer types, and the number of samples is given in the parentheses. The vertical axis lists the names of the genes.


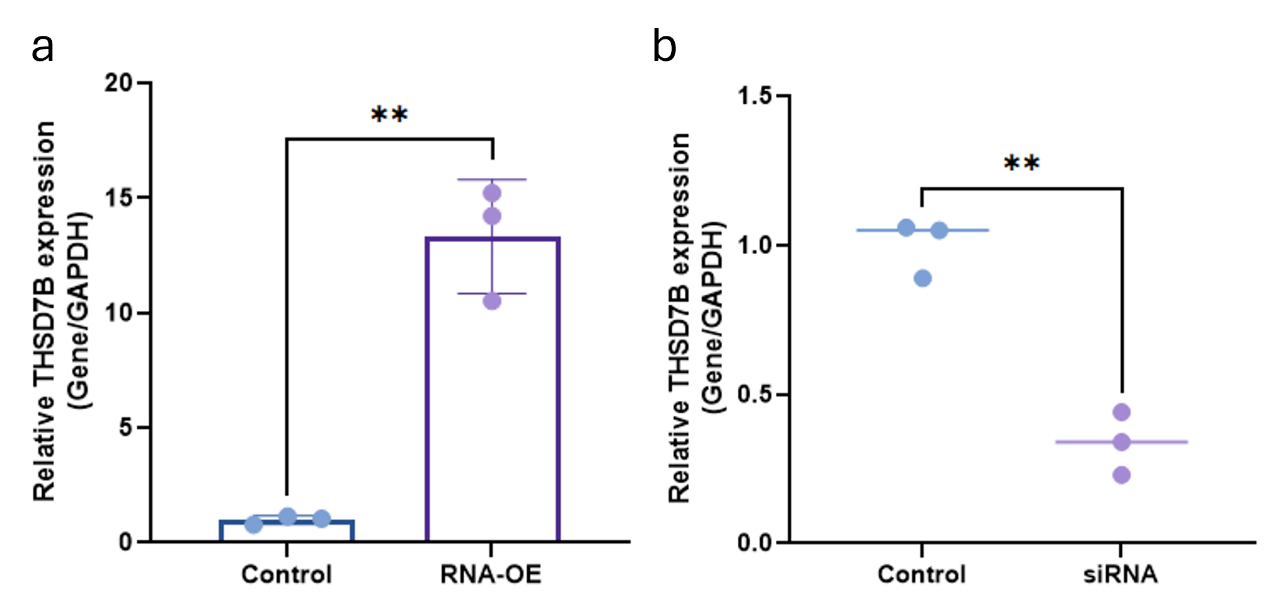


**Supplementary Figure 6**. (a-b) PCR validation of targeted gene. Significance was calculated with student t test. n = 3. ***P<0.001.

## Supplementary Tables

**Supplemental Table 1. Primers for PCR used.**

| Primer Name | Primers |
| --- | --- |
| H-THSD7B  NM_001316349.2  THSD7B （human）siRNA- 1946  THSD7B （human）siRNA- 1946 | F: ACAGTCACTTGCTGGGTATGG  R: AAAACACCACACTGCCCGA  GAGAGGAAGUCUUGUGAAAtt  UUUCACAAGACUUCCUCUCtt |
| GAPDH | F: GAAGGTGAAGGTCGGAGTCA  R: GACAAGCTTCCCGTTCTCAG |
